# Ethyl­enediammonium tetra­kis({2,2′-[ethane-1,2-diylbis(nitrilo­methyl­idyne)]diphenolato}nickel(II)) bis­(perchlorate) dimethyl­formamide monosolvate

**DOI:** 10.1107/S1600536810017162

**Published:** 2010-05-15

**Authors:** Gervas Assey, Yilma Gultneh, Ray J. Butcher

**Affiliations:** a Department of Chemistry, Howard University, 525 College Street NW, Washington, DC 20059, USA

## Abstract

The title compound, (C_2_H_10_N_2_)[Ni(C_16_H_14_N_2_O_2_)]_4_(ClO_4_)_2_·C_3_H_7_NO, crystallizes with four Ni(salen) mol­ecules {salen is 2,2′-[ethane-1,2-diylbis(nitrilo­methyl­idyne)]diphenolate}, one ethyl­enediammonium cation (actually two half-cations, each located on a center of inversion), two perchlorate anions and one dimethyl­formamide solvent mol­ecule in the asymmetric unit. Each Ni^II^ cation in the Ni(salen) complex is four-coordinated by two imine N atoms and two phenolate O atoms from the tetra­dentate ligand. The Ni(salen) units form parallel slipped stacks with Ni⋯Ni separations of 3.4541 (4) and 3.6442 (6) Å. The crystal packing is stabilized by inter­molecular hydrogen bonds between the ammonium H atoms and the perchlorate and salen O atoms, which generate a three-dimensional structure.

## Related literature

For applications of nickel–Schiff base complexes in homogeneous and heterogeneous catalysis, see: Santos *et al.* (2000 ▶); Silva *et al.* (2002 ▶); Yoon & Burrows (1988 ▶); Mitra & Chatterjee (1999 ▶). For other properties of Ni(salen) complexes, see: Abe *et al.* (2006 ▶); Gaetani Manfredotti & Guastini (1983 ▶); Pahor *et al.* (1976 ▶); Prabhakar *et al.* (2006 ▶); Santos *et al.* (2000 ▶); Silva *et al.* (2002 ▶). For the structures of Ni(salen) co-crystallization complexes, see: Giacomelli *et al.* (1982 ▶); Ryaza­nov *et al.* (2001 ▶); Skovsgaard *et al.* (2005 ▶); Feng *et al.* (2007 ▶); Sun *et al.* (1991 ▶); Lutz (2003 ▶).
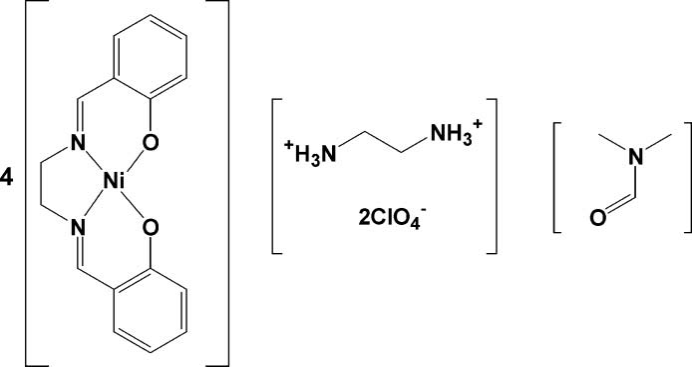


## Experimental

### 

#### Crystal data

(C_2_H_10_N_2_)[Ni(C_16_H_14_N_2_O_2_)]_4_·(ClO_4_)_2_·C_3_H_7_NO
*M* *_r_* = 1634.12
Triclinic, 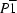

*a* = 15.0209 (11) Å
*b* = 15.0492 (13) Å
*c* = 18.2709 (8) Å
α = 85.990 (5)°
β = 86.506 (5)°
γ = 62.963 (8)°
*V* = 3667.8 (4) Å^3^
*Z* = 2
Mo *K*α radiation
μ = 1.16 mm^−1^
*T* = 200 K
0.53 × 0.28 × 0.24 mm

#### Data collection

Oxford Diffraction Gemini R diffractometer
Absorption correction: multi-scan (*CrysAlis RED*; Oxford Diffraction, 2007 ▶) *T*_min_ = 0.832, *T*_max_ = 1.000
31940 measured reflections
13062 independent reflections
9248 reflections with *I* > 2σ(*I*)
*R*_int_ = 0.031

#### Refinement

*R*[*F* ^2^ > 2σ(*F* ^2^)] = 0.039
*wR*(*F* ^2^) = 0.110
*S* = 0.95
13062 reflections
936 parameters
14 restraints
H-atom parameters constrained
Δρ_max_ = 0.56 e Å^−3^
Δρ_min_ = −0.32 e Å^−3^

### 

Data collection: *CrysAlis CCD* (Oxford Diffraction, 2007 ▶); cell refinement: *CrysAlis RED* (Oxford Diffraction, 2007 ▶); data reduction: *CrysAlis RED*; program(s) used to solve structure: *SHELXS97* (Sheldrick, 2008 ▶); program(s) used to refine structure: *SHELXL97* (Sheldrick, 2008 ▶); molecular graphics: *SHELXTL* (Sheldrick, 2008 ▶); software used to prepare material for publication: *SHELXTL*.

## Supplementary Material

Crystal structure: contains datablocks I, global. DOI: 10.1107/S1600536810017162/sj2787sup1.cif

Structure factors: contains datablocks I. DOI: 10.1107/S1600536810017162/sj2787Isup2.hkl

Additional supplementary materials:  crystallographic information; 3D view; checkCIF report

## Figures and Tables

**Table 1:** Hydrogen-bond geometry (Å, °)

*D*—H⋯*A*	*D*—H	H⋯*A*	*D*⋯*A*	*D*—H⋯*A*
N11*S*—H11*B*⋯O2*A*	0.91	1.97	2.877 (3)	173
N11*S*—H11*B*⋯O1*A*	0.91	2.45	2.949 (3)	115
N11*S*—H11*D*⋯O2*D*	0.91	2.00	2.876 (3)	162
N11*S*—H11*D*⋯O1*D*	0.91	2.48	3.132 (3)	128
N22*S*—H22*A*⋯O1*B*	0.91	1.97	2.830 (3)	157
N22*S*—H22*A*⋯O2*B*	0.91	2.34	2.884 (3)	118
N22*S*—H22*B*⋯O1*C*	0.91	1.98	2.853 (3)	160
N22*S*—H22*B*⋯O2*C*	0.91	2.45	3.118 (3)	130
N22*S*—H22*C*⋯O1*SB*	0.91	1.91	2.706 (5)	145
N22*S*—H22*C*⋯O1*SA*	0.91	1.90	2.778 (6)	161

**Fig. 1.**
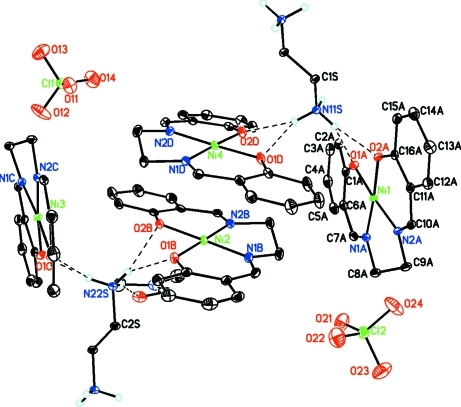
Showing the contents of the asymmetric unit with complete ethylenediammonium cations (generated by symmetry codes 1-x, 1-y, 1-z and 2-x, -y, 2-z). Hydrogen bonding between cations and perchlorate anions, neutral Ni(salen), and N,N'-dimethylformamide solvate molecule is shown by dashed lines. For clarity only the Ni, Cl O and N (from diammonium cations) atoms are labeled.

**Fig. 2.**
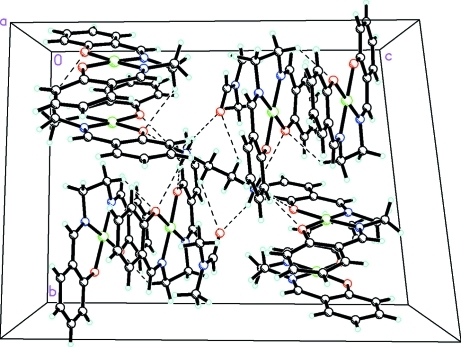
The molecular packing for C69H73Cl2N11Ni4O17 viewed down the a axis showing the intermolecular N—H···O interactions.

## supplementary crystallographic information

### Comment

Schiff base complexes of transition metals are of paramount importance due to their application in homogeneous and heterogeneous catalysis (Santos *et al.* 2000; Silva *et al.* 2002). Nickel(II) Schiff bases have been described as being active catalytically in oxidation and reduction reactions both as homogeneous (Yoon & Burrows 1988) and heterogeneous catalysts (Mitra & Chatterjee 1999). [Ethylenebis(salicylideneiminato-κ^4^*N*,*N*',O,*O*']nickel(II) {Nisalen} complexes with mesomorphic properties, known as metallomesogens, have been synthesized and investigated (Abe *et al.* 2006). In addition, there have been several instances of Ni(salen) type species co-crystallizing with other salts and molecules (Giacomelli *et al.*, 1982; Ryazanov *et al.*, 2001; Skovsgaard *et al.*, 2005; Feng *et al.*, 2007; Sun *et al.*, 1991; Lutz, 2003).

The work presented here describes the synthesis and structural characterization of Ni^II^ salen complex co-crystallizing with ethylenediammonium perchlorate and *N*,*N*'-dimethylformamide presented in Fig. 1. The asymmetric unit contains four neutral Ni(salen) molecules, ethylenediammonium perchlorate (actually two half ethylenediammonium cations lying on centers of inversion), and an *N*,*N*'-dimethylformamide solvate molecule. All species are linked together by an extensive series of hydrogen bonds between the ethylenediammonium cations and perchlorate anions, neutral Ni(salen) and *N*,*N*'-dimethylformamide molecules. This is a good example of molecular recognition. The Ni—O phenolate bond distances range from 1.8353 (15) to 1.8576 (14) Å and the Ni—N distances range from 1.836 (2) to 1.8497 (18) Å and are comparable to those found in literature for similar neutral Ni(salen) complexes reported earlier (Prabhakar *et al.* 2006). The coordination around Ni^II^ ions shows a slightly distorted square planer geometry. The Ni(salen) units form parallel slipped stacks with Ni—Ni separations of 3.4541 (4) and 3.6442 (6) Å.

### Experimental

The ligand ethylenebis(salicylideneimine) was synthesized by reacting a solution of (5 g, 83.19 mmol) of ethylenediamine in 10 ml ethanol with a solution of (20.32 g, 166.38 mmol) salicylaldehyde in 40 ml ethanol. The mixture was refluxed for 24 hrs. The mixture was then evaporated under reduced pressure and yellow solids were obtained with a yield of 96.6%.

The complex was synthesized by reacting 1.36 g (3.73 mmol) of Ni(ClO_4_)_2_.6H_2_O in methanol (10 ml) with 1 g (3.73 mmol) of ethylenebis(salicylideneimine) in CH_2_Cl_2_ (10 ml) for 24 hours while stirring with magnetic stirrer at room temperature. The mixture was evaporated under reduced pressure and brownish solids were obtained. These solids were dissolved in *N*,*N*'-dimethylformamide. The solution obtained was filtered and layered with diethyl ether. Brownish X-ray quality crystals were obtained after slow diffusion of the diethyl ether into the *N*,*N*'-dimethylformamide solution of the complex over a period of several days.

### Refinement

One DMF molecule was disordered in a manner that was not possible to model successfully. This was removed using the SQUEEZE routine from Platon. The output files from Platon are appended to the cif file and the fcf file has been modified using the Calc-FCF routine from Platon. H atoms were placed in geometrically idealized positions and constrained to ride on their parent atoms with a C—H distance of 0.95 and 0.99 Å *U*_iso_(H) = 1.2*U*_eq_(C) and 0.98 Å for CH_3_ [*U*_iso_(H) = 1.5*U*_eq_(C)]. The H atoms attached to N were idealized with an N–H distance of 0.91 Å.

### Crystal data

**Table:**

(C_2_H_10_N_2_)[Ni(C_16_H_14_N_2_O_2_)]_4_·(ClO_4_)_2_·C_3_H_7_NO	*Z* = 2
*M**_r_* = 1634.12	*F*(000) = 1692
Triclinic, *P*1	*D*_x_ = 1.480 Mg m^−^^3^
Hall symbol: -P 1	Mo *K*α radiation, λ = 0.71073 Å
*a* = 15.0209 (11) Å	Cell parameters from 14342 reflections
*b* = 15.0492 (13) Å	θ = 4.5–34.7°
*c* = 18.2709 (8) Å	µ = 1.16 mm^−^^1^
α = 85.990 (5)°	*T* = 200 K
β = 86.506 (5)°	Thick needle, translucent red-brown
γ = 62.963 (8)°	0.53 × 0.28 × 0.24 mm
*V* = 3667.8 (4) Å^3^	

### Data collection

**Table:**

Oxford Diffraction Gemini R diffractometer	13062 independent reflections
Radiation source: Enhance (Mo) X-ray Source	9248 reflections with *I* > 2σ(*I*)
graphite	*R*_int_ = 0.031
Detector resolution: 10.5081 pixels mm^-1^	θ_max_ = 25.2°, θ_min_ = 4.5°
ω scans	*h* = −18→18
Absorption correction: multi-scan (*CrysAlis RED*; Oxford Diffraction, 2007)	*k* = −17→18
*T*_min_ = 0.832, *T*_max_ = 1.000	*l* = −21→20
31940 measured reflections	

### Refinement

**Table:**

Refinement on *F*^2^	Primary atom site location: structure-invariant direct methods
Least-squares matrix: full	Secondary atom site location: difference Fourier map
*R*[*F*^2^ > 2σ(*F*^2^)] = 0.039	Hydrogen site location: inferred from neighbouring sites
*wR*(*F*^2^) = 0.110	H-atom parameters constrained
*S* = 0.95	*w* = 1/[σ^2^(*F*_o_^2^) + (0.0701*P*)^2^] where *P* = (*F*_o_^2^ + 2*F*_c_^2^)/3
13062 reflections	(Δ/σ)_max_ = 0.002
936 parameters	Δρ_max_ = 0.56 e Å^−^^3^
14 restraints	Δρ_min_ = −0.32 e Å^−^^3^

### Special details

**Table:**

Geometry. All esds (except the esd in the dihedral angle between two l.s. planes) are estimated using the full covariance matrix. The cell esds are taken into account individually in the estimation of esds in distances, angles and torsion angles; correlations between esds in cell parameters are only used when they are defined by crystal symmetry. An approximate (isotropic) treatment of cell esds is used for estimating esds involving l.s. planes.
Refinement. Refinement of *F*^2^ against ALL reflections. The weighted *R*-factor wR and goodness of fit *S* are based on *F*^2^, conventional *R*-factors *R* are based on *F*, with *F* set to zero for negative *F*^2^. The threshold expression of *F*^2^ > σ(*F*^2^) is used only for calculating *R*-factors(gt) etc. and is not relevant to the choice of reflections for refinement. *R*-factors based on *F*^2^ are statistically about twice as large as those based on *F*, and *R*- factors based on ALL data will be even larger.

### Fractional atomic coordinates and isotropic or equivalent isotropic displacement parameters (Å^2^)

**Table:**

	*x*	*y*	*z*	*U*_iso_*/*U*_eq_	Occ. (<1)
Ni1	0.36564 (2)	0.67261 (2)	0.77292 (2)	0.02687 (10)	
Ni2	0.76060 (3)	0.25911 (3)	0.82558 (2)	0.03289 (11)	
Ni3	1.11277 (3)	−0.12736 (3)	0.75047 (2)	0.02913 (10)	
Ni4	0.71105 (3)	0.29506 (3)	0.64077 (2)	0.03068 (10)	
Cl1	1.18947 (6)	0.10251 (6)	0.48440 (5)	0.0453 (2)	
Cl2	0.29975 (6)	0.41268 (6)	1.01193 (5)	0.0489 (2)	
O11	1.11501 (19)	0.0697 (2)	0.49421 (18)	0.0781 (9)	
O12	1.2601 (3)	0.0623 (3)	0.5382 (2)	0.1211 (14)	
O13	1.2386 (3)	0.0697 (3)	0.4153 (2)	0.1121 (13)	
O14	1.1438 (3)	0.2069 (2)	0.4780 (2)	0.1056 (12)	
O21	0.3826 (2)	0.4327 (2)	0.9983 (2)	0.0895 (10)	
O22	0.3318 (3)	0.3094 (2)	1.0094 (2)	0.1025 (12)	
O23	0.2627 (3)	0.4388 (3)	1.0858 (2)	0.1168 (13)	
O24	0.2220 (3)	0.4681 (3)	0.9663 (2)	0.1207 (14)	
O1A	0.44445 (14)	0.70365 (14)	0.70528 (11)	0.0323 (5)	
O2A	0.32163 (13)	0.63858 (13)	0.69153 (10)	0.0278 (4)	
O1B	0.78134 (13)	0.13008 (14)	0.85054 (12)	0.0361 (5)	
O2B	0.88915 (14)	0.19722 (13)	0.78735 (11)	0.0338 (5)	
O1C	1.16007 (13)	−0.08894 (14)	0.82778 (11)	0.0319 (5)	
O2C	1.03643 (14)	−0.15448 (14)	0.82302 (11)	0.0348 (5)	
O1D	0.58011 (14)	0.35649 (13)	0.67428 (12)	0.0365 (5)	
O2D	0.69133 (14)	0.42452 (14)	0.61834 (12)	0.0360 (5)	
N1A	0.41689 (17)	0.69773 (17)	0.85393 (13)	0.0331 (6)	
N2A	0.27928 (17)	0.64977 (17)	0.83877 (13)	0.0321 (6)	
N1B	0.63144 (18)	0.31786 (19)	0.86463 (15)	0.0415 (7)	
N2B	0.74371 (19)	0.38617 (17)	0.80112 (14)	0.0378 (6)	
N1C	1.19653 (17)	−0.10758 (16)	0.67991 (13)	0.0312 (6)	
N2C	1.05854 (17)	−0.15817 (16)	0.67420 (13)	0.0324 (6)	
N1D	0.73038 (18)	0.16699 (17)	0.66650 (14)	0.0336 (6)	
N2D	0.84188 (18)	0.23644 (17)	0.60470 (14)	0.0357 (6)	
N11S	0.48787 (17)	0.56377 (17)	0.58756 (13)	0.0322 (6)	
H11B	0.4390	0.5844	0.6235	0.048*	
H11C	0.4961	0.6170	0.5681	0.048*	
H11D	0.5462	0.5173	0.6069	0.048*	
N22S	0.97904 (17)	0.04318 (17)	0.90083 (13)	0.0318 (6)	
H22A	0.9244	0.0630	0.8730	0.048*	
H22B	1.0315	−0.0097	0.8805	0.048*	
H22C	0.9950	0.0945	0.9028	0.048*	
C1A	0.50091 (19)	0.74663 (19)	0.71817 (17)	0.0311 (7)	
C2A	0.5383 (2)	0.7840 (2)	0.6580 (2)	0.0391 (8)	
H2AA	0.5212	0.7794	0.6097	0.047*	
C3A	0.5994 (2)	0.8273 (2)	0.6684 (2)	0.0480 (9)	
H3AA	0.6250	0.8510	0.6269	0.058*	
C4A	0.6247 (2)	0.8372 (2)	0.7380 (2)	0.0507 (10)	
H4AA	0.6669	0.8676	0.7442	0.061*	
C5A	0.5885 (2)	0.8029 (2)	0.7970 (2)	0.0474 (9)	
H5AA	0.6062	0.8090	0.8448	0.057*	
C6A	0.5251 (2)	0.7581 (2)	0.78923 (19)	0.0361 (8)	
C7A	0.4821 (2)	0.7316 (2)	0.85369 (18)	0.0367 (8)	
H7AA	0.5030	0.7394	0.8998	0.044*	
C8A	0.3795 (2)	0.6719 (2)	0.92469 (18)	0.0437 (8)	
H8AA	0.3773	0.7166	0.9628	0.052*	
H8AB	0.4238	0.6021	0.9409	0.052*	
C9A	0.2767 (2)	0.6846 (2)	0.91269 (18)	0.0447 (8)	
H9AA	0.2583	0.6445	0.9502	0.054*	
H9AB	0.2270	0.7556	0.9158	0.054*	
C10A	0.2171 (2)	0.6167 (2)	0.82453 (17)	0.0349 (7)	
H10A	0.1788	0.6067	0.8646	0.042*	
C11A	0.20118 (19)	0.5937 (2)	0.75343 (17)	0.0308 (7)	
C12A	0.1293 (2)	0.5591 (2)	0.74596 (19)	0.0390 (8)	
H12A	0.0956	0.5482	0.7887	0.047*	
C13A	0.1070 (2)	0.5411 (2)	0.6799 (2)	0.0446 (8)	
H13A	0.0582	0.5180	0.6766	0.053*	
C14A	0.1555 (2)	0.5565 (2)	0.6168 (2)	0.0434 (8)	
H14A	0.1405	0.5432	0.5703	0.052*	
C15A	0.2259 (2)	0.5911 (2)	0.62179 (18)	0.0343 (7)	
H15A	0.2573	0.6033	0.5782	0.041*	
C16A	0.25146 (19)	0.60843 (19)	0.68946 (16)	0.0272 (6)	
C1B	0.7138 (2)	0.1011 (2)	0.87479 (17)	0.0356 (7)	
C2B	0.7414 (2)	−0.0013 (2)	0.87984 (19)	0.0444 (8)	
H2BA	0.8079	−0.0472	0.8664	0.053*	
C3B	0.6748 (3)	−0.0372 (3)	0.9038 (2)	0.0501 (9)	
H3BA	0.6959	−0.1072	0.9070	0.060*	
C4B	0.5765 (3)	0.0284 (3)	0.9234 (2)	0.0593 (10)	
H4BA	0.5300	0.0037	0.9387	0.071*	
C5B	0.5481 (2)	0.1284 (3)	0.92040 (19)	0.0531 (10)	
H5BA	0.4818	0.1729	0.9357	0.064*	
C6B	0.6145 (2)	0.1683 (3)	0.89507 (18)	0.0409 (8)	
C7B	0.5794 (2)	0.2739 (3)	0.89050 (18)	0.0451 (9)	
H7BA	0.5130	0.3145	0.9077	0.054*	
C8B	0.5881 (2)	0.4281 (2)	0.8668 (2)	0.0540 (10)	
H8BA	0.6000	0.4469	0.9148	0.065*	
H8BB	0.5151	0.4592	0.8599	0.065*	
C9B	0.6376 (2)	0.4631 (2)	0.8065 (2)	0.0494 (9)	
H9BA	0.6044	0.4711	0.7596	0.059*	
H9BB	0.6337	0.5283	0.8176	0.059*	
C10B	0.8120 (2)	0.4119 (2)	0.78000 (17)	0.0397 (8)	
H10B	0.7935	0.4813	0.7742	0.048*	
C11B	0.9148 (2)	0.3429 (2)	0.76451 (17)	0.0357 (7)	
C12B	0.9835 (3)	0.3799 (2)	0.7430 (2)	0.0483 (9)	
H12B	0.9624	0.4497	0.7437	0.058*	
C13B	1.0798 (3)	0.3185 (3)	0.7212 (2)	0.0573 (10)	
H13B	1.1251	0.3450	0.7066	0.069*	
C14B	1.1098 (2)	0.2167 (3)	0.7208 (2)	0.0535 (10)	
H14B	1.1764	0.1735	0.7053	0.064*	
C15B	1.0457 (2)	0.1767 (2)	0.74222 (19)	0.0426 (8)	
H15B	1.0682	0.1067	0.7411	0.051*	
C16B	0.9465 (2)	0.2391 (2)	0.76587 (16)	0.0327 (7)	
C1C	1.2291 (2)	−0.0573 (2)	0.82443 (17)	0.0309 (7)	
C2C	1.2578 (2)	−0.0351 (2)	0.89003 (19)	0.0405 (8)	
H2CA	1.2271	−0.0435	0.9352	0.049*	
C3C	1.3286 (2)	−0.0019 (2)	0.8903 (2)	0.0454 (9)	
H3CA	1.3465	0.0124	0.9354	0.054*	
C4C	1.3751 (2)	0.0113 (2)	0.8242 (2)	0.0458 (9)	
H4CA	1.4237	0.0352	0.8242	0.055*	
C5C	1.3495 (2)	−0.0105 (2)	0.7602 (2)	0.0411 (8)	
H5CA	1.3812	−0.0018	0.7156	0.049*	
C6C	1.2775 (2)	−0.0456 (2)	0.75791 (17)	0.0325 (7)	
C7C	1.2592 (2)	−0.0733 (2)	0.68950 (18)	0.0354 (8)	
H7CA	1.2962	−0.0658	0.6475	0.042*	
C8C	1.1948 (2)	−0.1447 (2)	0.60766 (17)	0.0388 (8)	
H8CA	1.2463	−0.2149	0.6043	0.047*	
H8CB	1.2086	−0.1039	0.5680	0.047*	
C9C	1.0926 (2)	−0.1371 (2)	0.60039 (17)	0.0398 (8)	
H9CA	1.0460	−0.0692	0.5817	0.048*	
H9CB	1.0953	−0.1860	0.5657	0.048*	
C10C	0.9931 (2)	−0.1919 (2)	0.67934 (18)	0.0363 (8)	
H10C	0.9691	−0.2011	0.6350	0.044*	
C11C	0.9543 (2)	−0.2164 (2)	0.74688 (18)	0.0350 (7)	
C12C	0.8921 (2)	−0.2642 (2)	0.7440 (2)	0.0446 (9)	
H12C	0.8713	−0.2722	0.6979	0.053*	
C13C	0.8619 (2)	−0.2986 (3)	0.8070 (2)	0.0518 (9)	
H13C	0.8205	−0.3308	0.8048	0.062*	
C14C	0.8919 (2)	−0.2866 (2)	0.8738 (2)	0.0468 (9)	
H14C	0.8717	−0.3119	0.9174	0.056*	
C15C	0.9501 (2)	−0.2387 (2)	0.87894 (19)	0.0416 (8)	
H15C	0.9691	−0.2309	0.9258	0.050*	
C16C	0.9819 (2)	−0.2011 (2)	0.81519 (17)	0.0314 (7)	
C1D	0.5271 (2)	0.3129 (2)	0.70293 (17)	0.0334 (7)	
C2D	0.4297 (2)	0.3737 (2)	0.7293 (2)	0.0488 (9)	
H2DA	0.4051	0.4441	0.7264	0.059*	
C3D	0.3693 (2)	0.3333 (2)	0.7590 (2)	0.0582 (11)	
H3DA	0.3034	0.3763	0.7760	0.070*	
C4D	0.4031 (3)	0.2306 (3)	0.7645 (2)	0.0551 (10)	
H4DA	0.3605	0.2032	0.7843	0.066*	
C5D	0.4985 (2)	0.1698 (2)	0.74121 (19)	0.0440 (8)	
H5DA	0.5224	0.0994	0.7457	0.053*	
C6D	0.5617 (2)	0.2087 (2)	0.71094 (17)	0.0339 (7)	
C7D	0.6643 (2)	0.1403 (2)	0.69302 (17)	0.0352 (7)	
H7DA	0.6844	0.0709	0.7014	0.042*	
C8D	0.8360 (2)	0.0903 (2)	0.66086 (19)	0.0417 (8)	
H8DA	0.8401	0.0252	0.6492	0.050*	
H8DB	0.8696	0.0818	0.7077	0.050*	
C9D	0.8844 (3)	0.1272 (2)	0.6003 (2)	0.0499 (9)	
H9DA	0.9576	0.0957	0.6061	0.060*	
H9DB	0.8709	0.1104	0.5521	0.060*	
C10D	0.8955 (2)	0.2802 (2)	0.58442 (18)	0.0397 (8)	
H10D	0.9623	0.2398	0.5679	0.048*	
C11D	0.8623 (2)	0.3857 (2)	0.58476 (17)	0.0356 (7)	
C12D	0.9321 (2)	0.4235 (3)	0.56833 (18)	0.0440 (8)	
H12D	0.9992	0.3788	0.5552	0.053*	
C13D	0.9052 (2)	0.5231 (3)	0.5709 (2)	0.0479 (9)	
H13D	0.9532	0.5471	0.5599	0.058*	
C14D	0.8064 (2)	0.5895 (3)	0.5900 (2)	0.0470 (9)	
H14D	0.7874	0.6588	0.5917	0.056*	
C15D	0.7365 (2)	0.5548 (2)	0.6064 (2)	0.0437 (8)	
H15D	0.6700	0.6005	0.6201	0.052*	
C16D	0.7620 (2)	0.4527 (2)	0.60309 (17)	0.0341 (7)	
C1S	0.4589 (2)	0.5194 (2)	0.52937 (16)	0.0315 (7)	
H1SA	0.3965	0.5704	0.5072	0.038*	
H1SB	0.4462	0.4639	0.5510	0.038*	
C2S	0.9568 (2)	0.0137 (2)	0.97578 (16)	0.0308 (7)	
H2SA	0.9424	−0.0441	0.9739	0.037*	
H2SB	0.8967	0.0696	0.9966	0.037*	
O1SA	1.0125 (5)	0.2006 (2)	0.9393 (4)	0.0541 (12)	0.475 (7)
O1SB	0.9653 (4)	0.2151 (3)	0.9490 (4)	0.0541 (12)	0.525 (7)
C11S	0.9732 (4)	0.2955 (2)	0.9377 (2)	0.0765 (13)	
H11A	0.9066	0.3474	0.9401	0.092*	
N1S	1.0477 (2)	0.3206 (2)	0.92681 (17)	0.0540 (8)	
C12S	1.1467 (4)	0.2466 (4)	0.9204 (3)	0.118 (2)	
H12E	1.1793	0.2363	0.9673	0.176*	
H12F	1.1826	0.2676	0.8820	0.176*	
H12G	1.1476	0.1841	0.9075	0.176*	
C13S	1.0265 (4)	0.4245 (3)	0.9169 (3)	0.0888 (15)	
H13E	0.9539	0.4666	0.9190	0.133*	
H13F	1.0539	0.4357	0.8690	0.133*	
H13G	1.0571	0.4417	0.9559	0.133*	

### Atomic displacement parameters (Å^2^)

**Table:**

	*U*^11^	*U*^22^	*U*^33^	*U*^12^	*U*^13^	*U*^23^
Ni1	0.02955 (18)	0.02476 (18)	0.0227 (2)	−0.00915 (14)	0.00090 (15)	−0.00292 (14)
Ni2	0.02913 (19)	0.0295 (2)	0.0315 (2)	−0.00472 (15)	−0.00538 (16)	−0.00590 (16)
Ni3	0.03140 (19)	0.02646 (19)	0.0248 (2)	−0.00951 (15)	0.00294 (15)	−0.00093 (15)
Ni4	0.0333 (2)	0.02499 (19)	0.0293 (2)	−0.00892 (15)	−0.00515 (16)	−0.00059 (15)
Cl1	0.0452 (4)	0.0433 (4)	0.0488 (6)	−0.0217 (4)	−0.0073 (4)	0.0049 (4)
Cl2	0.0408 (4)	0.0520 (5)	0.0502 (6)	−0.0171 (4)	−0.0040 (4)	−0.0049 (4)
O11	0.0602 (16)	0.0778 (19)	0.107 (3)	−0.0428 (14)	−0.0024 (16)	0.0130 (17)
O12	0.118 (3)	0.154 (3)	0.116 (3)	−0.086 (2)	−0.075 (2)	0.070 (3)
O13	0.102 (2)	0.153 (3)	0.095 (3)	−0.070 (2)	0.037 (2)	−0.041 (2)
O14	0.134 (3)	0.0545 (18)	0.134 (3)	−0.0464 (19)	−0.021 (2)	0.0071 (19)
O21	0.0608 (17)	0.103 (2)	0.118 (3)	−0.0481 (16)	0.0177 (17)	−0.027 (2)
O22	0.105 (2)	0.066 (2)	0.130 (3)	−0.0320 (18)	−0.011 (2)	−0.014 (2)
O23	0.118 (3)	0.162 (4)	0.077 (3)	−0.068 (3)	0.030 (2)	−0.048 (2)
O24	0.093 (2)	0.139 (3)	0.132 (3)	−0.059 (2)	−0.061 (2)	0.076 (3)
O1A	0.0360 (10)	0.0373 (11)	0.0285 (12)	−0.0204 (9)	0.0020 (9)	−0.0066 (9)
O2A	0.0288 (9)	0.0321 (10)	0.0238 (11)	−0.0151 (8)	0.0030 (8)	−0.0031 (8)
O1B	0.0269 (10)	0.0337 (11)	0.0439 (14)	−0.0103 (9)	−0.0014 (9)	−0.0032 (9)
O2B	0.0331 (10)	0.0254 (10)	0.0350 (13)	−0.0062 (8)	−0.0036 (9)	0.0001 (9)
O1C	0.0324 (10)	0.0363 (11)	0.0294 (12)	−0.0183 (9)	0.0065 (8)	−0.0035 (9)
O2C	0.0400 (11)	0.0403 (11)	0.0283 (12)	−0.0221 (9)	0.0053 (9)	−0.0042 (9)
O1D	0.0300 (10)	0.0235 (10)	0.0518 (15)	−0.0086 (8)	−0.0063 (9)	0.0041 (9)
O2D	0.0326 (10)	0.0282 (10)	0.0455 (14)	−0.0127 (9)	−0.0048 (9)	0.0051 (9)
N1A	0.0379 (13)	0.0276 (12)	0.0246 (15)	−0.0060 (11)	−0.0024 (11)	−0.0049 (10)
N2A	0.0347 (13)	0.0295 (13)	0.0217 (14)	−0.0061 (11)	0.0021 (10)	0.0000 (10)
N1B	0.0335 (14)	0.0420 (15)	0.0373 (17)	−0.0049 (12)	−0.0054 (12)	−0.0121 (12)
N2B	0.0392 (14)	0.0293 (13)	0.0321 (16)	−0.0029 (11)	−0.0074 (12)	−0.0078 (11)
N1C	0.0338 (13)	0.0237 (12)	0.0261 (15)	−0.0052 (10)	0.0032 (10)	0.0021 (10)
N2C	0.0340 (13)	0.0271 (12)	0.0272 (15)	−0.0060 (11)	−0.0025 (11)	0.0006 (10)
N1D	0.0379 (13)	0.0252 (12)	0.0295 (15)	−0.0057 (11)	−0.0063 (11)	−0.0065 (10)
N2D	0.0421 (14)	0.0286 (13)	0.0287 (15)	−0.0094 (11)	0.0019 (11)	−0.0029 (10)
N11S	0.0326 (12)	0.0297 (13)	0.0267 (15)	−0.0077 (10)	0.0023 (10)	−0.0027 (10)
N22S	0.0355 (13)	0.0332 (13)	0.0229 (14)	−0.0124 (10)	−0.0020 (10)	0.0013 (10)
C1A	0.0261 (14)	0.0214 (14)	0.041 (2)	−0.0056 (11)	−0.0022 (13)	−0.0060 (12)
C2A	0.0344 (16)	0.0355 (17)	0.050 (2)	−0.0178 (13)	0.0037 (14)	−0.0075 (15)
C3A	0.0347 (17)	0.0365 (18)	0.071 (3)	−0.0149 (14)	0.0022 (17)	−0.0028 (17)
C4A	0.0356 (17)	0.0396 (19)	0.081 (3)	−0.0187 (15)	−0.0092 (18)	−0.0079 (18)
C5A	0.0396 (17)	0.0381 (18)	0.064 (3)	−0.0144 (15)	−0.0191 (17)	−0.0086 (17)
C6A	0.0290 (15)	0.0259 (15)	0.047 (2)	−0.0062 (12)	−0.0081 (14)	−0.0041 (13)
C7A	0.0389 (17)	0.0272 (15)	0.036 (2)	−0.0063 (13)	−0.0111 (14)	−0.0071 (13)
C8A	0.0526 (19)	0.0417 (18)	0.027 (2)	−0.0127 (15)	−0.0002 (15)	−0.0046 (14)
C9A	0.054 (2)	0.0471 (19)	0.0242 (19)	−0.0162 (16)	0.0075 (15)	−0.0059 (14)
C10A	0.0305 (15)	0.0321 (16)	0.0310 (19)	−0.0067 (13)	0.0085 (13)	0.0062 (13)
C11A	0.0250 (14)	0.0246 (14)	0.0352 (19)	−0.0060 (11)	0.0032 (12)	0.0057 (12)
C12A	0.0314 (15)	0.0357 (17)	0.047 (2)	−0.0148 (13)	0.0014 (14)	0.0102 (14)
C13A	0.0422 (18)	0.0420 (18)	0.056 (3)	−0.0252 (15)	−0.0061 (16)	0.0049 (16)
C14A	0.0361 (16)	0.0398 (18)	0.057 (2)	−0.0183 (14)	−0.0085 (16)	−0.0038 (16)
C15A	0.0279 (14)	0.0371 (16)	0.038 (2)	−0.0143 (13)	0.0032 (13)	−0.0059 (13)
C16A	0.0216 (13)	0.0200 (13)	0.0347 (18)	−0.0051 (11)	0.0001 (12)	−0.0008 (12)
C1B	0.0317 (15)	0.0520 (19)	0.0260 (18)	−0.0206 (14)	−0.0047 (13)	−0.0059 (14)
C2B	0.0406 (17)	0.051 (2)	0.044 (2)	−0.0222 (15)	−0.0009 (15)	−0.0049 (16)
C3B	0.051 (2)	0.065 (2)	0.046 (2)	−0.0371 (18)	−0.0023 (17)	−0.0017 (17)
C4B	0.054 (2)	0.097 (3)	0.045 (2)	−0.051 (2)	−0.0020 (18)	0.002 (2)
C5B	0.0351 (17)	0.089 (3)	0.034 (2)	−0.0273 (18)	0.0042 (15)	−0.0066 (19)
C6B	0.0299 (16)	0.061 (2)	0.0275 (19)	−0.0162 (15)	−0.0038 (13)	−0.0067 (15)
C7B	0.0284 (16)	0.062 (2)	0.032 (2)	−0.0070 (16)	−0.0002 (14)	−0.0162 (16)
C8B	0.0418 (18)	0.0427 (19)	0.057 (3)	0.0018 (15)	−0.0020 (17)	−0.0231 (17)
C9B	0.0430 (18)	0.0317 (17)	0.053 (2)	0.0040 (14)	−0.0117 (17)	−0.0122 (16)
C10B	0.056 (2)	0.0290 (16)	0.0245 (18)	−0.0103 (15)	−0.0087 (15)	−0.0022 (13)
C11B	0.0517 (18)	0.0281 (15)	0.0231 (18)	−0.0142 (14)	−0.0095 (14)	0.0043 (12)
C12B	0.064 (2)	0.0368 (18)	0.047 (2)	−0.0262 (17)	−0.0061 (17)	0.0059 (15)
C13B	0.055 (2)	0.047 (2)	0.072 (3)	−0.0275 (18)	−0.0059 (19)	0.0167 (18)
C14B	0.0376 (18)	0.045 (2)	0.070 (3)	−0.0146 (16)	−0.0005 (17)	0.0157 (18)
C15B	0.0387 (17)	0.0283 (16)	0.051 (2)	−0.0081 (14)	−0.0042 (15)	0.0097 (14)
C16B	0.0385 (16)	0.0353 (16)	0.0223 (17)	−0.0150 (13)	−0.0078 (13)	0.0052 (12)
C1C	0.0254 (14)	0.0233 (14)	0.0359 (19)	−0.0044 (12)	0.0032 (12)	−0.0022 (12)
C2C	0.0358 (16)	0.0450 (18)	0.040 (2)	−0.0185 (14)	0.0101 (14)	−0.0078 (15)
C3C	0.0369 (17)	0.0448 (19)	0.055 (2)	−0.0176 (15)	−0.0015 (16)	−0.0109 (16)
C4C	0.0317 (16)	0.0359 (17)	0.069 (3)	−0.0158 (14)	0.0042 (16)	0.0007 (16)
C5C	0.0358 (16)	0.0335 (17)	0.050 (2)	−0.0139 (14)	0.0048 (15)	0.0070 (15)
C6C	0.0298 (15)	0.0253 (15)	0.0337 (19)	−0.0061 (12)	0.0053 (13)	0.0012 (12)
C7C	0.0302 (15)	0.0292 (15)	0.0332 (19)	−0.0037 (13)	0.0084 (13)	0.0061 (13)
C8C	0.0447 (18)	0.0357 (17)	0.0241 (18)	−0.0092 (14)	0.0056 (14)	0.0030 (13)
C9C	0.0533 (19)	0.0350 (17)	0.0220 (18)	−0.0123 (14)	−0.0014 (14)	0.0017 (13)
C10C	0.0333 (16)	0.0311 (16)	0.033 (2)	−0.0040 (13)	−0.0077 (13)	−0.0014 (13)
C11C	0.0272 (14)	0.0311 (16)	0.039 (2)	−0.0065 (12)	−0.0022 (13)	−0.0008 (13)
C12C	0.0343 (16)	0.0451 (19)	0.052 (2)	−0.0145 (14)	−0.0083 (15)	−0.0058 (16)
C13C	0.0356 (17)	0.052 (2)	0.070 (3)	−0.0225 (15)	−0.0047 (17)	−0.0007 (19)
C14C	0.0377 (17)	0.051 (2)	0.052 (2)	−0.0225 (16)	0.0038 (16)	0.0060 (17)
C15C	0.0421 (17)	0.0460 (19)	0.037 (2)	−0.0211 (15)	0.0006 (15)	0.0024 (15)
C16C	0.0269 (14)	0.0285 (15)	0.0332 (19)	−0.0081 (12)	0.0008 (12)	−0.0003 (12)
C1D	0.0313 (15)	0.0290 (15)	0.0382 (19)	−0.0118 (13)	−0.0102 (13)	0.0039 (13)
C2D	0.0343 (17)	0.0295 (16)	0.076 (3)	−0.0098 (14)	−0.0055 (17)	0.0095 (16)
C3D	0.0358 (18)	0.0370 (19)	0.091 (3)	−0.0095 (15)	0.0011 (19)	0.0100 (19)
C4D	0.0455 (19)	0.046 (2)	0.077 (3)	−0.0253 (16)	−0.0047 (18)	0.0129 (18)
C5D	0.0504 (19)	0.0275 (16)	0.052 (2)	−0.0163 (15)	−0.0045 (16)	0.0045 (14)
C6D	0.0383 (16)	0.0272 (15)	0.0341 (19)	−0.0124 (13)	−0.0088 (13)	0.0007 (13)
C7D	0.0465 (18)	0.0261 (15)	0.0324 (19)	−0.0154 (14)	−0.0041 (14)	−0.0035 (13)
C8D	0.0435 (18)	0.0221 (15)	0.046 (2)	−0.0028 (13)	−0.0007 (15)	−0.0055 (14)
C9D	0.056 (2)	0.0298 (17)	0.050 (2)	−0.0084 (15)	0.0124 (17)	−0.0093 (15)
C10D	0.0374 (16)	0.0412 (18)	0.032 (2)	−0.0103 (14)	0.0022 (14)	−0.0039 (14)
C11D	0.0334 (15)	0.0426 (18)	0.0268 (18)	−0.0139 (13)	−0.0043 (13)	0.0023 (13)
C12D	0.0378 (17)	0.059 (2)	0.035 (2)	−0.0218 (16)	−0.0031 (14)	0.0017 (16)
C13D	0.0461 (19)	0.060 (2)	0.047 (2)	−0.0328 (17)	−0.0051 (16)	0.0046 (17)
C14D	0.054 (2)	0.0451 (19)	0.051 (2)	−0.0312 (16)	−0.0081 (17)	0.0043 (16)
C15D	0.0365 (17)	0.0363 (17)	0.054 (2)	−0.0134 (14)	−0.0057 (15)	0.0028 (15)
C16D	0.0375 (16)	0.0353 (16)	0.0300 (19)	−0.0169 (13)	−0.0087 (13)	0.0056 (13)
C1S	0.0299 (14)	0.0375 (16)	0.0252 (17)	−0.0135 (12)	0.0040 (12)	−0.0057 (12)
C2S	0.0329 (15)	0.0367 (16)	0.0245 (17)	−0.0183 (12)	0.0012 (12)	0.0039 (12)
O1SA	0.079 (3)	0.0598 (16)	0.053 (2)	−0.0575 (18)	−0.008 (2)	0.0071 (14)
O1SB	0.079 (3)	0.0598 (16)	0.053 (2)	−0.0575 (18)	−0.008 (2)	0.0071 (14)
C11S	0.120 (4)	0.085 (3)	0.037 (3)	−0.058 (3)	−0.024 (2)	0.008 (2)
N1S	0.077 (2)	0.0476 (17)	0.050 (2)	−0.0389 (16)	−0.0076 (16)	−0.0001 (14)
C12S	0.104 (4)	0.112 (4)	0.082 (4)	0.002 (3)	−0.002 (3)	−0.027 (3)
C13S	0.129 (4)	0.069 (3)	0.081 (4)	−0.055 (3)	0.004 (3)	−0.012 (2)

### Geometric parameters (Å, °)

**Table:**

Ni1—O1A	1.841 (2)	C5B—H5BA	0.9500
Ni1—N2A	1.845 (3)	C6B—C7B	1.427 (5)
Ni1—N1A	1.846 (2)	C7B—H7BA	0.9500
Ni1—O2A	1.8561 (19)	C8B—C9B	1.489 (5)
Ni1—Ni3^i^	3.6441 (6)	C8B—H8BA	0.9900
Ni2—N2B	1.837 (3)	C8B—H8BB	0.9900
Ni2—O2B	1.8371 (19)	C9B—H9BA	0.9900
Ni2—N1B	1.848 (3)	C9B—H9BB	0.9900
Ni2—O1B	1.849 (2)	C10B—C11B	1.440 (4)
Ni2—Ni4	3.4544 (6)	C10B—H10B	0.9500
Ni3—N2C	1.842 (3)	C11B—C12B	1.404 (5)
Ni3—O2C	1.845 (2)	C11B—C16B	1.409 (4)
Ni3—N1C	1.850 (2)	C12B—C13B	1.369 (5)
Ni3—O1C	1.856 (2)	C12B—H12B	0.9500
Ni4—O1D	1.8377 (19)	C13B—C14B	1.387 (5)
Ni4—N1D	1.844 (2)	C13B—H13B	0.9500
Ni4—N2D	1.850 (2)	C14B—C15B	1.376 (5)
Ni4—O2D	1.8519 (19)	C14B—H14B	0.9500
Cl1—O12	1.385 (3)	C15B—C16B	1.412 (4)
Cl1—O14	1.399 (3)	C15B—H15B	0.9500
Cl1—O11	1.411 (3)	C1C—C2C	1.408 (4)
Cl1—O13	1.424 (4)	C1C—C6C	1.420 (4)
Cl2—O24	1.375 (3)	C2C—C3C	1.365 (5)
Cl2—O22	1.406 (3)	C2C—H2CA	0.9500
Cl2—O21	1.412 (3)	C3C—C4C	1.406 (5)
Cl2—O23	1.438 (4)	C3C—H3CA	0.9500
O1A—C1A	1.318 (3)	C4C—C5C	1.358 (5)
O2A—C16A	1.327 (3)	C4C—H4CA	0.9500
O1B—C1B	1.318 (4)	C5C—C6C	1.405 (4)
O2B—C16B	1.309 (4)	C5C—H5CA	0.9500
O1C—C1C	1.320 (3)	C6C—C7C	1.425 (4)
O2C—C16C	1.317 (4)	C7C—H7CA	0.9500
O1D—C1D	1.309 (4)	C8C—C9C	1.500 (5)
O2D—C16D	1.320 (4)	C8C—H8CA	0.9900
N1A—C7A	1.293 (4)	C8C—H8CB	0.9900
N1A—C8A	1.476 (4)	C9C—H9CA	0.9900
N2A—C10A	1.287 (4)	C9C—H9CB	0.9900
N2A—C9A	1.475 (4)	C10C—C11C	1.430 (4)
N1B—C7B	1.287 (4)	C10C—H10C	0.9500
N1B—C8B	1.484 (4)	C11C—C16C	1.405 (4)
N2B—C10B	1.282 (4)	C11C—C12C	1.419 (5)
N2B—C9B	1.487 (4)	C12C—C13C	1.367 (5)
N1C—C7C	1.287 (4)	C12C—H12C	0.9500
N1C—C8C	1.475 (4)	C13C—C14C	1.378 (5)
N2C—C10C	1.291 (4)	C13C—H13C	0.9500
N2C—C9C	1.478 (4)	C14C—C15C	1.372 (5)
N1D—C7D	1.287 (4)	C14C—H14C	0.9500
N1D—C8D	1.481 (4)	C15C—C16C	1.412 (4)
N2D—C10D	1.278 (4)	C15C—H15C	0.9500
N2D—C9D	1.474 (4)	C1D—C2D	1.403 (4)
N11S—C1S	1.478 (4)	C1D—C6D	1.410 (4)
N11S—H11B	0.9100	C2D—C3D	1.372 (5)
N11S—H11C	0.9100	C2D—H2DA	0.9500
N11S—H11D	0.9100	C3D—C4D	1.388 (5)
N22S—C2S	1.476 (4)	C3D—H3DA	0.9500
N22S—H22A	0.9100	C4D—C5D	1.365 (5)
N22S—H22B	0.9100	C4D—H4DA	0.9500
N22S—H22C	0.9100	C5D—C6D	1.395 (5)
C1A—C2A	1.402 (4)	C5D—H5DA	0.9500
C1A—C6A	1.412 (4)	C6D—C7D	1.444 (4)
C2A—C3A	1.374 (5)	C7D—H7DA	0.9500
C2A—H2AA	0.9500	C8D—C9D	1.497 (5)
C3A—C4A	1.385 (5)	C8D—H8DA	0.9900
C3A—H3AA	0.9500	C8D—H8DB	0.9900
C4A—C5A	1.354 (5)	C9D—H9DA	0.9900
C4A—H4AA	0.9500	C9D—H9DB	0.9900
C5A—C6A	1.412 (5)	C10D—C11D	1.432 (4)
C5A—H5AA	0.9500	C10D—H10D	0.9500
C6A—C7A	1.428 (5)	C11D—C12D	1.411 (5)
C7A—H7AA	0.9500	C11D—C16D	1.416 (4)
C8A—C9A	1.496 (5)	C12D—C13D	1.367 (5)
C8A—H8AA	0.9900	C12D—H12D	0.9500
C8A—H8AB	0.9900	C13D—C14D	1.399 (5)
C9A—H9AA	0.9900	C13D—H13D	0.9500
C9A—H9AB	0.9900	C14D—C15D	1.381 (5)
C10A—C11A	1.428 (4)	C14D—H14D	0.9500
C10A—H10A	0.9500	C15D—C16D	1.408 (4)
C11A—C16A	1.412 (4)	C15D—H15D	0.9500
C11A—C12A	1.413 (4)	C1S—C1S^ii^	1.512 (5)
C12A—C13A	1.348 (5)	C1S—H1SA	0.9900
C12A—H12A	0.9500	C1S—H1SB	0.9900
C13A—C14A	1.388 (5)	C2S—C2S^iii^	1.497 (5)
C13A—H13A	0.9500	C2S—H2SA	0.9900
C14A—C15A	1.385 (4)	C2S—H2SB	0.9900
C14A—H14A	0.9500	O1SA—C11S	1.2720 (10)
C15A—C16A	1.392 (4)	O1SB—C11S	1.2699 (10)
C15A—H15A	0.9500	C11S—N1S	1.335 (5)
C1B—C2B	1.400 (4)	C11S—H11A	0.9500
C1B—C6B	1.413 (4)	N1S—C12S	1.399 (5)
C2B—C3B	1.374 (5)	N1S—C13S	1.447 (5)
C2B—H2BA	0.9500	C12S—H12E	0.9800
C3B—C4B	1.394 (5)	C12S—H12F	0.9800
C3B—H3BA	0.9500	C12S—H12G	0.9800
C4B—C5B	1.363 (5)	C13S—H13E	0.9800
C4B—H4BA	0.9500	C13S—H13F	0.9800
C5B—C6B	1.421 (5)	C13S—H13G	0.9800
			
O1A—Ni1—N2A	176.03 (9)	C9B—C8B—H8BB	110.2
O1A—Ni1—N1A	95.23 (10)	H8BA—C8B—H8BB	108.5
N2A—Ni1—N1A	85.89 (12)	N2B—C9B—C8B	107.0 (3)
O1A—Ni1—O2A	84.32 (8)	N2B—C9B—H9BA	110.3
N2A—Ni1—O2A	94.83 (10)	C8B—C9B—H9BA	110.3
N1A—Ni1—O2A	176.02 (9)	N2B—C9B—H9BB	110.3
O1A—Ni1—Ni3^i^	105.44 (6)	C8B—C9B—H9BB	110.3
N2A—Ni1—Ni3^i^	70.59 (7)	H9BA—C9B—H9BB	108.6
N1A—Ni1—Ni3^i^	107.78 (7)	N2B—C10B—C11B	124.5 (3)
O2A—Ni1—Ni3^i^	76.13 (5)	N2B—C10B—H10B	117.8
N2B—Ni2—O2B	94.75 (10)	C11B—C10B—H10B	117.8
N2B—Ni2—N1B	86.87 (12)	C12B—C11B—C16B	119.2 (3)
O2B—Ni2—N1B	178.38 (11)	C12B—C11B—C10B	119.4 (3)
N2B—Ni2—O1B	178.41 (10)	C16B—C11B—C10B	121.3 (3)
O2B—Ni2—O1B	83.90 (9)	C13B—C12B—C11B	122.0 (3)
N1B—Ni2—O1B	94.49 (11)	C13B—C12B—H12B	119.0
N2B—Ni2—Ni4	75.14 (8)	C11B—C12B—H12B	119.0
O2B—Ni2—Ni4	80.61 (6)	C12B—C13B—C14B	118.5 (3)
N1B—Ni2—Ni4	99.72 (8)	C12B—C13B—H13B	120.7
O1B—Ni2—Ni4	105.42 (7)	C14B—C13B—H13B	120.7
N2C—Ni3—O2C	95.11 (10)	C15B—C14B—C13B	121.7 (3)
N2C—Ni3—N1C	86.16 (11)	C15B—C14B—H14B	119.2
O2C—Ni3—N1C	176.24 (9)	C13B—C14B—H14B	119.2
N2C—Ni3—O1C	176.42 (9)	C14B—C15B—C16B	120.3 (3)
O2C—Ni3—O1C	83.96 (9)	C14B—C15B—H15B	119.8
N1C—Ni3—O1C	94.99 (10)	C16B—C15B—H15B	119.8
O1D—Ni4—N1D	95.37 (10)	O2B—C16B—C11B	123.8 (3)
O1D—Ni4—N2D	178.16 (10)	O2B—C16B—C15B	118.0 (3)
N1D—Ni4—N2D	86.17 (11)	C11B—C16B—C15B	118.2 (3)
O1D—Ni4—O2D	83.91 (9)	O1C—C1C—C2C	118.8 (3)
N1D—Ni4—O2D	178.02 (10)	O1C—C1C—C6C	123.4 (3)
N2D—Ni4—O2D	94.58 (10)	C2C—C1C—C6C	117.8 (3)
O1D—Ni4—Ni2	83.51 (7)	C3C—C2C—C1C	121.7 (3)
N1D—Ni4—Ni2	74.24 (8)	C3C—C2C—H2CA	119.2
N2D—Ni4—Ni2	97.88 (8)	C1C—C2C—H2CA	119.2
O2D—Ni4—Ni2	103.84 (7)	C2C—C3C—C4C	120.4 (3)
O12—Cl1—O14	113.8 (2)	C2C—C3C—H3CA	119.8
O12—Cl1—O11	112.2 (2)	C4C—C3C—H3CA	119.8
O14—Cl1—O11	109.2 (2)	C5C—C4C—C3C	119.1 (3)
O12—Cl1—O13	108.4 (3)	C5C—C4C—H4CA	120.4
O14—Cl1—O13	105.7 (2)	C3C—C4C—H4CA	120.4
O11—Cl1—O13	107.1 (2)	C4C—C5C—C6C	122.1 (3)
O24—Cl2—O22	112.7 (2)	C4C—C5C—H5CA	119.0
O24—Cl2—O21	113.1 (2)	C6C—C5C—H5CA	119.0
O22—Cl2—O21	109.3 (2)	C5C—C6C—C1C	119.0 (3)
O24—Cl2—O23	107.0 (3)	C5C—C6C—C7C	119.0 (3)
O22—Cl2—O23	106.3 (3)	C1C—C6C—C7C	121.9 (3)
O21—Cl2—O23	108.2 (2)	N1C—C7C—C6C	125.3 (3)
C1A—O1A—Ni1	126.6 (2)	N1C—C7C—H7CA	117.3
C16A—O2A—Ni1	127.90 (18)	C6C—C7C—H7CA	117.3
C1B—O1B—Ni2	127.18 (18)	N1C—C8C—C9C	107.3 (2)
C16B—O2B—Ni2	127.14 (17)	N1C—C8C—H8CA	110.3
C1C—O1C—Ni3	127.25 (19)	C9C—C8C—H8CA	110.3
C16C—O2C—Ni3	126.25 (19)	N1C—C8C—H8CB	110.3
C1D—O1D—Ni4	126.93 (17)	C9C—C8C—H8CB	110.3
C16D—O2D—Ni4	126.05 (17)	H8CA—C8C—H8CB	108.5
C7A—N1A—C8A	119.3 (3)	N2C—C9C—C8C	107.2 (3)
C7A—N1A—Ni1	126.7 (2)	N2C—C9C—H9CA	110.3
C8A—N1A—Ni1	114.0 (2)	C8C—C9C—H9CA	110.3
C10A—N2A—C9A	119.1 (3)	N2C—C9C—H9CB	110.3
C10A—N2A—Ni1	126.9 (2)	C8C—C9C—H9CB	110.3
C9A—N2A—Ni1	113.7 (2)	H9CA—C9C—H9CB	108.5
C7B—N1B—C8B	119.3 (3)	N2C—C10C—C11C	124.8 (3)
C7B—N1B—Ni2	127.4 (2)	N2C—C10C—H10C	117.6
C8B—N1B—Ni2	113.3 (2)	C11C—C10C—H10C	117.6
C10B—N2B—C9B	119.9 (3)	C16C—C11C—C12C	119.7 (3)
C10B—N2B—Ni2	126.9 (2)	C16C—C11C—C10C	121.7 (3)
C9B—N2B—Ni2	113.2 (2)	C12C—C11C—C10C	118.4 (3)
C7C—N1C—C8C	119.0 (3)	C13C—C12C—C11C	120.5 (3)
C7C—N1C—Ni3	126.9 (2)	C13C—C12C—H12C	119.7
C8C—N1C—Ni3	113.9 (2)	C11C—C12C—H12C	119.7
C10C—N2C—C9C	118.7 (3)	C12C—C13C—C14C	119.7 (3)
C10C—N2C—Ni3	126.8 (2)	C12C—C13C—H13C	120.2
C9C—N2C—Ni3	114.4 (2)	C14C—C13C—H13C	120.2
C7D—N1D—C8D	118.6 (3)	C15C—C14C—C13C	121.5 (3)
C7D—N1D—Ni4	127.1 (2)	C15C—C14C—H14C	119.3
C8D—N1D—Ni4	114.1 (2)	C13C—C14C—H14C	119.3
C10D—N2D—C9D	119.6 (3)	C14C—C15C—C16C	120.5 (3)
C10D—N2D—Ni4	127.1 (2)	C14C—C15C—H15C	119.7
C9D—N2D—Ni4	113.3 (2)	C16C—C15C—H15C	119.7
C1S—N11S—H11B	109.5	O2C—C16C—C11C	123.8 (3)
C1S—N11S—H11C	109.5	O2C—C16C—C15C	118.3 (3)
H11B—N11S—H11C	109.5	C11C—C16C—C15C	118.0 (3)
C1S—N11S—H11D	109.5	O1D—C1D—C2D	118.1 (3)
H11B—N11S—H11D	109.5	O1D—C1D—C6D	124.7 (3)
H11C—N11S—H11D	109.5	C2D—C1D—C6D	117.2 (3)
C2S—N22S—H22A	109.5	C3D—C2D—C1D	121.3 (3)
C2S—N22S—H22B	109.5	C3D—C2D—H2DA	119.3
H22A—N22S—H22B	109.5	C1D—C2D—H2DA	119.3
C2S—N22S—H22C	109.5	C2D—C3D—C4D	120.9 (3)
H22A—N22S—H22C	109.5	C2D—C3D—H3DA	119.6
H22B—N22S—H22C	109.5	C4D—C3D—H3DA	119.6
O1A—C1A—C2A	118.2 (3)	C5D—C4D—C3D	119.0 (3)
O1A—C1A—C6A	123.7 (3)	C5D—C4D—H4DA	120.5
C2A—C1A—C6A	118.1 (3)	C3D—C4D—H4DA	120.5
C3A—C2A—C1A	120.4 (3)	C4D—C5D—C6D	121.4 (3)
C3A—C2A—H2AA	119.8	C4D—C5D—H5DA	119.3
C1A—C2A—H2AA	119.8	C6D—C5D—H5DA	119.3
C2A—C3A—C4A	121.7 (4)	C5D—C6D—C1D	120.1 (3)
C2A—C3A—H3AA	119.2	C5D—C6D—C7D	118.6 (3)
C4A—C3A—H3AA	119.2	C1D—C6D—C7D	121.2 (3)
C5A—C4A—C3A	119.0 (3)	N1D—C7D—C6D	124.5 (3)
C5A—C4A—H4AA	120.5	N1D—C7D—H7DA	117.8
C3A—C4A—H4AA	120.5	C6D—C7D—H7DA	117.8
C4A—C5A—C6A	121.5 (4)	N1D—C8D—C9D	106.2 (2)
C4A—C5A—H5AA	119.2	N1D—C8D—H8DA	110.5
C6A—C5A—H5AA	119.2	C9D—C8D—H8DA	110.5
C1A—C6A—C5A	119.3 (3)	N1D—C8D—H8DB	110.5
C1A—C6A—C7A	121.8 (3)	C9D—C8D—H8DB	110.5
C5A—C6A—C7A	118.8 (3)	H8DA—C8D—H8DB	108.7
N1A—C7A—C6A	124.9 (3)	N2D—C9D—C8D	107.6 (2)
N1A—C7A—H7AA	117.6	N2D—C9D—H9DA	110.2
C6A—C7A—H7AA	117.6	C8D—C9D—H9DA	110.2
N1A—C8A—C9A	106.9 (3)	N2D—C9D—H9DB	110.2
N1A—C8A—H8AA	110.3	C8D—C9D—H9DB	110.2
C9A—C8A—H8AA	110.3	H9DA—C9D—H9DB	108.5
N1A—C8A—H8AB	110.3	N2D—C10D—C11D	124.8 (3)
C9A—C8A—H8AB	110.3	N2D—C10D—H10D	117.6
H8AA—C8A—H8AB	108.6	C11D—C10D—H10D	117.6
N2A—C9A—C8A	106.4 (2)	C12D—C11D—C16D	119.2 (3)
N2A—C9A—H9AA	110.4	C12D—C11D—C10D	119.1 (3)
C8A—C9A—H9AA	110.4	C16D—C11D—C10D	121.7 (3)
N2A—C9A—H9AB	110.4	C13D—C12D—C11D	121.4 (3)
C8A—C9A—H9AB	110.4	C13D—C12D—H12D	119.3
H9AA—C9A—H9AB	108.6	C11D—C12D—H12D	119.3
N2A—C10A—C11A	125.4 (3)	C12D—C13D—C14D	119.7 (3)
N2A—C10A—H10A	117.3	C12D—C13D—H13D	120.1
C11A—C10A—H10A	117.3	C14D—C13D—H13D	120.1
C16A—C11A—C12A	118.4 (3)	C15D—C14D—C13D	120.2 (3)
C16A—C11A—C10A	122.5 (3)	C15D—C14D—H14D	119.9
C12A—C11A—C10A	119.1 (3)	C13D—C14D—H14D	119.9
C13A—C12A—C11A	122.0 (3)	C14D—C15D—C16D	121.2 (3)
C13A—C12A—H12A	119.0	C14D—C15D—H15D	119.4
C11A—C12A—H12A	119.0	C16D—C15D—H15D	119.4
C12A—C13A—C14A	119.9 (3)	O2D—C16D—C15D	118.2 (3)
C12A—C13A—H13A	120.1	O2D—C16D—C11D	123.5 (3)
C14A—C13A—H13A	120.1	C15D—C16D—C11D	118.3 (3)
C15A—C14A—C13A	119.9 (3)	N11S—C1S—C1S^ii^	110.3 (3)
C15A—C14A—H14A	120.0	N11S—C1S—H1SA	109.6
C13A—C14A—H14A	120.0	C1S^ii^—C1S—H1SA	109.6
C14A—C15A—C16A	121.2 (3)	N11S—C1S—H1SB	109.6
C14A—C15A—H15A	119.4	C1S^ii^—C1S—H1SB	109.6
C16A—C15A—H15A	119.4	H1SA—C1S—H1SB	108.1
O2A—C16A—C15A	118.9 (3)	N22S—C2S—C2S^iii^	110.8 (3)
O2A—C16A—C11A	122.4 (3)	N22S—C2S—H2SA	109.5
C15A—C16A—C11A	118.6 (3)	C2S^iii^—C2S—H2SA	109.5
O1B—C1B—C2B	118.3 (3)	N22S—C2S—H2SB	109.5
O1B—C1B—C6B	123.3 (3)	C2S^iii^—C2S—H2SB	109.5
C2B—C1B—C6B	118.4 (3)	H2SA—C2S—H2SB	108.1
C3B—C2B—C1B	121.7 (3)	O1SB—C11S—O1SA	30.0 (3)
C3B—C2B—H2BA	119.1	O1SB—C11S—N1S	136.5 (4)
C1B—C2B—H2BA	119.1	O1SA—C11S—N1S	106.9 (5)
C2B—C3B—C4B	120.4 (4)	O1SB—C11S—H11A	105.0
C2B—C3B—H3BA	119.8	O1SA—C11S—H11A	134.6
C4B—C3B—H3BA	119.8	N1S—C11S—H11A	118.4
C5B—C4B—C3B	119.2 (4)	C11S—N1S—C12S	120.3 (4)
C5B—C4B—H4BA	120.4	C11S—N1S—C13S	120.3 (3)
C3B—C4B—H4BA	120.4	C12S—N1S—C13S	119.3 (4)
C4B—C5B—C6B	121.9 (3)	N1S—C12S—H12E	109.5
C4B—C5B—H5BA	119.0	N1S—C12S—H12F	109.5
C6B—C5B—H5BA	119.0	H12E—C12S—H12F	109.5
C1B—C6B—C5B	118.4 (3)	N1S—C12S—H12G	109.5
C1B—C6B—C7B	122.2 (3)	H12E—C12S—H12G	109.5
C5B—C6B—C7B	119.4 (3)	H12F—C12S—H12G	109.5
N1B—C7B—C6B	124.5 (3)	N1S—C13S—H13E	109.5
N1B—C7B—H7BA	117.8	N1S—C13S—H13F	109.5
C6B—C7B—H7BA	117.8	H13E—C13S—H13F	109.5
N1B—C8B—C9B	107.7 (3)	N1S—C13S—H13G	109.5
N1B—C8B—H8BA	110.2	H13E—C13S—H13G	109.5
C9B—C8B—H8BA	110.2	H13F—C13S—H13G	109.5
N1B—C8B—H8BB	110.2		
			
N2B—Ni2—Ni4—O1D	−83.41 (11)	C10A—C11A—C12A—C13A	176.5 (3)
O2B—Ni2—Ni4—O1D	179.08 (9)	C11A—C12A—C13A—C14A	0.1 (5)
N1B—Ni2—Ni4—O1D	0.68 (11)	C12A—C13A—C14A—C15A	−0.6 (5)
O1B—Ni2—Ni4—O1D	98.13 (9)	C13A—C14A—C15A—C16A	1.9 (4)
N2B—Ni2—Ni4—N1D	179.11 (12)	Ni1—O2A—C16A—C15A	176.85 (17)
O2B—Ni2—Ni4—N1D	81.61 (10)	Ni1—O2A—C16A—C11A	−2.8 (3)
N1B—Ni2—Ni4—N1D	−96.79 (12)	C14A—C15A—C16A—O2A	177.9 (2)
O1B—Ni2—Ni4—N1D	0.66 (10)	C14A—C15A—C16A—C11A	−2.5 (4)
N2B—Ni2—Ni4—N2D	95.37 (12)	C12A—C11A—C16A—O2A	−178.5 (2)
O2B—Ni2—Ni4—N2D	−2.13 (10)	C10A—C11A—C16A—O2A	4.4 (4)
N1B—Ni2—Ni4—N2D	179.47 (12)	C12A—C11A—C16A—C15A	1.9 (4)
O1B—Ni2—Ni4—N2D	−83.08 (10)	C10A—C11A—C16A—C15A	−175.3 (2)
N2B—Ni2—Ni4—O2D	−1.35 (10)	Ni2—O1B—C1B—C2B	170.2 (2)
O2B—Ni2—Ni4—O2D	−98.85 (9)	Ni2—O1B—C1B—C6B	−9.0 (4)
N1B—Ni2—Ni4—O2D	82.75 (11)	O1B—C1B—C2B—C3B	−179.2 (3)
O1B—Ni2—Ni4—O2D	−179.80 (8)	C6B—C1B—C2B—C3B	0.0 (5)
N2A—Ni1—O1A—C1A	95.6 (15)	C1B—C2B—C3B—C4B	0.5 (5)
N1A—Ni1—O1A—C1A	−10.7 (2)	C2B—C3B—C4B—C5B	−1.7 (5)
O2A—Ni1—O1A—C1A	173.3 (2)	C3B—C4B—C5B—C6B	2.5 (5)
Ni3^i^—Ni1—O1A—C1A	99.4 (2)	O1B—C1B—C6B—C5B	179.9 (3)
O1A—Ni1—O2A—C16A	−176.6 (2)	C2B—C1B—C6B—C5B	0.7 (4)
N2A—Ni1—O2A—C16A	−0.5 (2)	O1B—C1B—C6B—C7B	0.3 (5)
N1A—Ni1—O2A—C16A	99.8 (15)	C2B—C1B—C6B—C7B	−178.9 (3)
Ni3^i^—Ni1—O2A—C16A	−69.09 (18)	C4B—C5B—C6B—C1B	−2.0 (5)
N2B—Ni2—O1B—C1B	159 (4)	C4B—C5B—C6B—C7B	177.6 (3)
O2B—Ni2—O1B—C1B	−169.1 (2)	C8B—N1B—C7B—C6B	−176.6 (3)
N1B—Ni2—O1B—C1B	10.8 (2)	Ni2—N1B—C7B—C6B	1.5 (5)
Ni4—Ni2—O1B—C1B	−90.6 (2)	C1B—C6B—C7B—N1B	3.6 (5)
N2B—Ni2—O2B—C16B	11.8 (2)	C5B—C6B—C7B—N1B	−176.0 (3)
N1B—Ni2—O2B—C16B	−172 (32)	C7B—N1B—C8B—C9B	−154.3 (3)
O1B—Ni2—O2B—C16B	−167.3 (2)	Ni2—N1B—C8B—C9B	27.4 (3)
Ni4—Ni2—O2B—C16B	85.9 (2)	C10B—N2B—C9B—C8B	−149.2 (3)
N2C—Ni3—O1C—C1C	−107.1 (16)	Ni2—N2B—C9B—C8B	31.8 (3)
O2C—Ni3—O1C—C1C	177.8 (2)	N1B—C8B—C9B—N2B	−36.3 (4)
N1C—Ni3—O1C—C1C	1.5 (2)	C9B—N2B—C10B—C11B	−172.3 (3)
N2C—Ni3—O2C—C16C	12.6 (2)	Ni2—N2B—C10B—C11B	6.6 (5)
N1C—Ni3—O2C—C16C	−97.0 (16)	N2B—C10B—C11B—C12B	−178.8 (3)
O1C—Ni3—O2C—C16C	−170.9 (2)	N2B—C10B—C11B—C16B	5.3 (5)
N1D—Ni4—O1D—C1D	−2.4 (3)	C16B—C11B—C12B—C13B	2.0 (5)
N2D—Ni4—O1D—C1D	145 (3)	C10B—C11B—C12B—C13B	−173.9 (3)
O2D—Ni4—O1D—C1D	179.5 (2)	C11B—C12B—C13B—C14B	−0.3 (6)
Ni2—Ni4—O1D—C1D	−75.8 (2)	C12B—C13B—C14B—C15B	−0.5 (6)
O1D—Ni4—O2D—C16D	164.4 (2)	C13B—C14B—C15B—C16B	−0.4 (6)
N1D—Ni4—O2D—C16D	96 (3)	Ni2—O2B—C16B—C11B	−4.3 (4)
N2D—Ni4—O2D—C16D	−16.7 (2)	Ni2—O2B—C16B—C15B	176.1 (2)
Ni2—Ni4—O2D—C16D	82.6 (2)	C12B—C11B—C16B—O2B	177.7 (3)
O1A—Ni1—N1A—C7A	3.5 (2)	C10B—C11B—C16B—O2B	−6.5 (5)
N2A—Ni1—N1A—C7A	−172.6 (2)	C12B—C11B—C16B—C15B	−2.8 (4)
O2A—Ni1—N1A—C7A	86.8 (15)	C10B—C11B—C16B—C15B	173.1 (3)
Ni3^i^—Ni1—N1A—C7A	−104.5 (2)	C14B—C15B—C16B—O2B	−178.4 (3)
O1A—Ni1—N1A—C8A	−174.27 (18)	C14B—C15B—C16B—C11B	2.0 (5)
N2A—Ni1—N1A—C8A	9.55 (19)	Ni3—O1C—C1C—C2C	−177.59 (18)
O2A—Ni1—N1A—C8A	−91.0 (15)	Ni3—O1C—C1C—C6C	1.6 (4)
Ni3^i^—Ni1—N1A—C8A	77.66 (18)	O1C—C1C—C2C—C3C	−179.8 (3)
O1A—Ni1—N2A—C10A	80.2 (15)	C6C—C1C—C2C—C3C	1.0 (4)
N1A—Ni1—N2A—C10A	−173.3 (2)	C1C—C2C—C3C—C4C	0.1 (5)
O2A—Ni1—N2A—C10A	2.8 (2)	C2C—C3C—C4C—C5C	−0.8 (5)
Ni3^i^—Ni1—N2A—C10A	76.2 (2)	C3C—C4C—C5C—C6C	0.3 (4)
O1A—Ni1—N2A—C9A	−92.8 (15)	C4C—C5C—C6C—C1C	0.8 (4)
N1A—Ni1—N2A—C9A	13.72 (19)	C4C—C5C—C6C—C7C	−175.9 (3)
O2A—Ni1—N2A—C9A	−170.21 (19)	O1C—C1C—C6C—C5C	179.4 (2)
Ni3^i^—Ni1—N2A—C9A	−96.76 (19)	C2C—C1C—C6C—C5C	−1.4 (4)
N2B—Ni2—N1B—C7B	173.8 (3)	O1C—C1C—C6C—C7C	−4.0 (4)
O2B—Ni2—N1B—C7B	−2(4)	C2C—C1C—C6C—C7C	175.2 (2)
O1B—Ni2—N1B—C7B	−7.1 (3)	C8C—N1C—C7C—C6C	−172.5 (2)
Ni4—Ni2—N1B—C7B	99.4 (3)	Ni3—N1C—C7C—C6C	1.4 (4)
N2B—Ni2—N1B—C8B	−8.1 (2)	C5C—C6C—C7C—N1C	179.0 (2)
O2B—Ni2—N1B—C8B	176 (100)	C1C—C6C—C7C—N1C	2.5 (4)
O1B—Ni2—N1B—C8B	171.1 (2)	C7C—N1C—C8C—C9C	−155.6 (2)
Ni4—Ni2—N1B—C8B	−82.4 (2)	Ni3—N1C—C8C—C9C	29.7 (3)
O2B—Ni2—N2B—C10B	−12.9 (3)	C10C—N2C—C9C—C8C	−154.9 (2)
N1B—Ni2—N2B—C10B	167.2 (3)	Ni3—N2C—C9C—C8C	27.5 (3)
O1B—Ni2—N2B—C10B	19 (4)	N1C—C8C—C9C—N2C	−34.8 (3)
Ni4—Ni2—N2B—C10B	−91.9 (3)	C9C—N2C—C10C—C11C	179.4 (2)
O2B—Ni2—N2B—C9B	166.0 (2)	Ni3—N2C—C10C—C11C	−3.3 (4)
N1B—Ni2—N2B—C9B	−13.8 (2)	N2C—C10C—C11C—C16C	2.9 (4)
O1B—Ni2—N2B—C9B	−162 (4)	N2C—C10C—C11C—C12C	−172.4 (3)
Ni4—Ni2—N2B—C9B	87.1 (2)	C16C—C11C—C12C—C13C	−2.4 (4)
N2C—Ni3—N1C—C7C	173.7 (2)	C10C—C11C—C12C—C13C	173.0 (3)
O2C—Ni3—N1C—C7C	−76.5 (16)	C11C—C12C—C13C—C14C	0.2 (5)
O1C—Ni3—N1C—C7C	−2.9 (2)	C12C—C13C—C14C—C15C	1.3 (5)
N2C—Ni3—N1C—C8C	−12.17 (18)	C13C—C14C—C15C—C16C	−0.6 (5)
O2C—Ni3—N1C—C8C	97.7 (16)	Ni3—O2C—C16C—C11C	−16.0 (4)
O1C—Ni3—N1C—C8C	171.23 (18)	Ni3—O2C—C16C—C15C	163.4 (2)
O2C—Ni3—N2C—C10C	−3.1 (2)	C12C—C11C—C16C—O2C	−177.6 (2)
N1C—Ni3—N2C—C10C	173.3 (2)	C10C—C11C—C16C—O2C	7.2 (4)
O1C—Ni3—N2C—C10C	−77.8 (17)	C12C—C11C—C16C—C15C	3.0 (4)
O2C—Ni3—N2C—C9C	174.25 (18)	C10C—C11C—C16C—C15C	−172.2 (3)
N1C—Ni3—N2C—C9C	−9.30 (18)	C14C—C15C—C16C—O2C	179.0 (3)
O1C—Ni3—N2C—C9C	99.6 (16)	C14C—C15C—C16C—C11C	−1.6 (4)
O1D—Ni4—N1D—C7D	5.0 (3)	Ni4—O1D—C1D—C2D	176.3 (2)
N2D—Ni4—N1D—C7D	−174.1 (3)	Ni4—O1D—C1D—C6D	−2.1 (4)
O2D—Ni4—N1D—C7D	74 (3)	O1D—C1D—C2D—C3D	179.0 (3)
Ni2—Ni4—N1D—C7D	86.6 (3)	C6D—C1D—C2D—C3D	−2.5 (5)
O1D—Ni4—N1D—C8D	−169.6 (2)	C1D—C2D—C3D—C4D	0.6 (6)
N2D—Ni4—N1D—C8D	11.4 (2)	C2D—C3D—C4D—C5D	1.2 (6)
O2D—Ni4—N1D—C8D	−101 (3)	C3D—C4D—C5D—C6D	−1.1 (6)
Ni2—Ni4—N1D—C8D	−87.9 (2)	C4D—C5D—C6D—C1D	−0.9 (5)
O1D—Ni4—N2D—C10D	44 (4)	C4D—C5D—C6D—C7D	174.9 (3)
N1D—Ni4—N2D—C10D	−168.4 (3)	O1D—C1D—C6D—C5D	−179.0 (3)
O2D—Ni4—N2D—C10D	9.7 (3)	C2D—C1D—C6D—C5D	2.6 (5)
Ni2—Ni4—N2D—C10D	−94.9 (3)	O1D—C1D—C6D—C7D	5.4 (5)
O1D—Ni4—N2D—C9D	−136 (3)	C2D—C1D—C6D—C7D	−173.1 (3)
N1D—Ni4—N2D—C9D	11.5 (2)	C8D—N1D—C7D—C6D	171.2 (3)
O2D—Ni4—N2D—C9D	−170.3 (2)	Ni4—N1D—C7D—C6D	−3.2 (4)
Ni2—Ni4—N2D—C9D	85.0 (2)	C5D—C6D—C7D—N1D	−178.3 (3)
Ni1—O1A—C1A—C2A	−166.28 (19)	C1D—C6D—C7D—N1D	−2.6 (5)
Ni1—O1A—C1A—C6A	13.0 (4)	C7D—N1D—C8D—C9D	154.6 (3)
O1A—C1A—C2A—C3A	−178.4 (2)	Ni4—N1D—C8D—C9D	−30.4 (3)
C6A—C1A—C2A—C3A	2.3 (4)	C10D—N2D—C9D—C8D	149.1 (3)
C1A—C2A—C3A—C4A	−1.2 (4)	Ni4—N2D—C9D—C8D	−30.9 (3)
C2A—C3A—C4A—C5A	0.3 (5)	N1D—C8D—C9D—N2D	37.4 (4)
C3A—C4A—C5A—C6A	−0.5 (5)	C9D—N2D—C10D—C11D	178.8 (3)
O1A—C1A—C6A—C5A	178.2 (2)	Ni4—N2D—C10D—C11D	−1.2 (5)
C2A—C1A—C6A—C5A	−2.5 (4)	N2D—C10D—C11D—C12D	173.4 (3)
O1A—C1A—C6A—C7A	−5.8 (4)	N2D—C10D—C11D—C16D	−4.9 (5)
C2A—C1A—C6A—C7A	173.5 (2)	C16D—C11D—C12D—C13D	1.1 (5)
C4A—C5A—C6A—C1A	1.7 (4)	C10D—C11D—C12D—C13D	−177.2 (3)
C4A—C5A—C6A—C7A	−174.4 (3)	C11D—C12D—C13D—C14D	−0.2 (5)
C8A—N1A—C7A—C6A	179.2 (2)	C12D—C13D—C14D—C15D	0.2 (5)
Ni1—N1A—C7A—C6A	1.5 (4)	C13D—C14D—C15D—C16D	−1.1 (5)
C1A—C6A—C7A—N1A	−1.8 (4)	Ni4—O2D—C16D—C15D	−163.5 (2)
C5A—C6A—C7A—N1A	174.2 (3)	Ni4—O2D—C16D—C11D	15.5 (4)
C7A—N1A—C8A—C9A	152.6 (3)	C14D—C15D—C16D—O2D	−179.0 (3)
Ni1—N1A—C8A—C9A	−29.5 (3)	C14D—C15D—C16D—C11D	2.0 (5)
C10A—N2A—C9A—C8A	153.8 (3)	C12D—C11D—C16D—O2D	179.1 (3)
Ni1—N2A—C9A—C8A	−32.6 (3)	C10D—C11D—C16D—O2D	−2.6 (5)
N1A—C8A—C9A—N2A	37.8 (3)	C12D—C11D—C16D—C15D	−1.9 (4)
C9A—N2A—C10A—C11A	170.7 (2)	C10D—C11D—C16D—C15D	176.3 (3)
Ni1—N2A—C10A—C11A	−1.9 (4)	O1SB—C11S—N1S—C12S	5.2 (8)
N2A—C10A—C11A—C16A	−2.0 (4)	O1SA—C11S—N1S—C12S	−0.6 (6)
N2A—C10A—C11A—C12A	−179.2 (3)	O1SB—C11S—N1S—C13S	−178.7 (6)
C16A—C11A—C12A—C13A	−0.8 (4)	O1SA—C11S—N1S—C13S	175.4 (5)

Symmetry codes: (i) *x*−1, *y*+1, *z*; (ii) −*x*+1, −*y*+1, −*z*+1; (iii) −*x*+2, −*y*, −*z*+2.

### Hydrogen-bond geometry (Å, °)

**Table:**

*D*—H···*A*	*D*—H	H···*A*	*D*···*A*	*D*—H···*A*
N11S—H11B···O2A	0.91	1.97	2.877 (3)	173
N11S—H11B···O1A	0.91	2.45	2.949 (3)	115
N11S—H11D···O2D	0.91	2.00	2.876 (3)	162
N11S—H11D···O1D	0.91	2.48	3.132 (3)	128
N22S—H22A···O1B	0.91	1.97	2.830 (3)	157
N22S—H22A···O2B	0.91	2.34	2.884 (3)	118
N22S—H22B···O1C	0.91	1.98	2.853 (3)	160
N22S—H22B···O2C	0.91	2.45	3.118 (3)	130
N22S—H22C···O1SB	0.91	1.91	2.706 (5)	145
N22S—H22C···O1SA	0.91	1.90	2.778 (6)	161
